# Unveiling Colorectal Cancer Biomarkers: Harnessing Biosensor Technology for Volatile Organic Compound Detection

**DOI:** 10.3390/s24144712

**Published:** 2024-07-20

**Authors:** Rebecca Golfinopoulou, Kyriaki Hatziagapiou, Sophie Mavrikou, Spyridon Kintzios

**Affiliations:** 1 Laboratory of Cell Technology, Department of Biotechnology, Agricultural University of Athens, EU-CONEXUS European University, 11855 Athens, Greece; skin@aua.gr; 2 First Department of Pediatrics, National and Kapodistrian University of Athens, “Aghia Sophia” Children’s Hospital, Thivon 1, 11527 Athens, Greece; khatziag@med.uoa.gr; 3 CeBTec, 40 Vatatzi, 11472 Athens, Greece

**Keywords:** biosensor, colorectal cancer, volatile organic compounds, VOC, early diagnosis, non-invasive, biomarkers

## Abstract

Conventional screening options for colorectal cancer (CRC) detection are mainly direct visualization and invasive methods including colonoscopy and flexible sigmoidoscopy, which must be performed in a clinical setting and may be linked to adverse effects for some patients. Non-invasive CRC diagnostic tests such as computed tomography colonography and stool tests are either too costly or less reliable than invasive ones. On the other hand, volatile organic compounds (VOCs) are potentially ideal non-invasive biomarkers for CRC detection and monitoring. The present review is a comprehensive presentation of the current state-of-the-art VOC-based CRC diagnostics, with a specific focus on recent advancements in biosensor design and application. Among them, breath-based chromatography pattern analysis and sampling techniques are overviewed, along with nanoparticle-based optical and electrochemical biosensor approaches. Limitations of the currently available technologies are also discussed with an outlook for improvement in combination with big data analytics and advanced instrumentation, as well as expanding the scope and specificity of CRC-related volatile biomarkers.

## 1. Introduction

Cancer is considered a major public health, societal, and economic challenge in the 21st century, accounting for almost one in four premature deaths (22.8%) from noncommunicable diseases (NCDs) worldwide [1,2]. Colorectal cancer (CRC) ranks third in terms of incidence (10%), following breast cancer, and lung cancer with an estimated 1.9 million new cases occurring in 2020 worldwide. When considering mortality, it is the second (9.4%) most common cause of death from cancer, with an estimated 935,173 deaths, following lung cancer [1]. Although the burden of CRC varies widely, more than 66% of all cases and 60% of all deaths occur in countries with a high or very high human development index (HDI). However, in the highest HDI countries, such as Japan, France, and the USA, there are decreases in both incidence and mortality, whereas in very high HDI-indexed countries, such as Canada, the UK, and Singapore increase in incidence trends for all ages is stabilized and combined with concomitant decline in mortality. Increases in the last decade in both incidence and mortality rates are observed in rapidly transitioning countries characterized by medium and high HDI, including Russia, China, and Brazil [1,3,4]. The adoption of a ‘Westernized’ lifestyle is mainly responsible for the global burden of CRC. These modifiable risk factors include poor dietary patterns, e.g., a relatively greater intake of red and processed meat, fat, and sugar and low consumption of whole grains, fruits, and vegetables, paralleled by sedentary behavior, alcohol consumption, smoking, and antibiotic usage, affecting the gut microbiome [4]. The lifetime risk of CRC in many Western regions is around 5%, with varying risks depending on geographical areas [5,6,7].

In high HDI countries, the decrease in CRC incidence and mortality rates from 2011 through 2019 by about 1% per year overall is attributed to a shift towards a healthier lifestyle with behavioral and dietary alterations. More importantly, they coincide with the advent of newer and better CRC screening tools and their widespread implementation, which have been established since the 1990s. The adoption of colonoscopy, flexible sigmoidoscopy, CT colonography, and fecal testing, although temporarily associated with increases in CRC incidence trends, due to the early detection of precursor lesions and asymptomatic disease, eventually contribute to mortality decrease in the forthcoming years due to the removal of precancerous polyps during colonoscopy [1,4]. CRC-related mortality was reduced by about 2.7% between 2004 and 2013 and is expected to further decline to about 38% for patients aged 50–74 years and 45% for patients > 75 years old by 2030 [8]. However, the declining incidence is restricted to individuals older than 65 years old and has stabilized in adults 55–64 years old. Surprisingly, there are recent studies suggesting a rise since the mid-1990s in CRC among adults younger than 55 years of age at diagnosis, with incidence rising by 1–2% per year. More importantly, in adults younger than 45 years at diagnosis, the incidence is rising by 1–4% per year [4]. A recent analysis demonstrated a concerning trend of a steep rise of 500% in CRC among younger populations in the U.S., and 333% in children aged 10–14 and 15–19, respectively, and by 185%, 71%, 58%, and 37% in young adults aged 20–24, 30–34, 35–39, and 40–44, respectively, highlighting that CRC is no longer considered a disease of the elderly population [9]. The same trend in younger ages is observed in Europe, as observed in national and regional cancer registries; CRC incidence increased by 7.9% per year from 2004 to 2016, in the age group of 20–29 years, 4.9% per year from 2005 to 2016 among individuals aged 30–39 years, and 1.6% per year from 2004 to 2016 in subjects 40–49 years [10,11]. Unfortunately, early-onset CRC is more likely to harbor more aggressive histopathology and is diagnosed at a more advanced stage, due to delay in seeking care or poor symptom evaluation from medical providers [10]. The burden of CRC is projected to surge by 60–140%, with more than 2.2 million new cases, and 1.1 million deaths by 2030; hence, integrating more effective, widespread, and robust screening in public health policies for individuals aged 45–75 years is the best preventive measure [12,13,14,15,16].

Considering the prohibitive costs of colonoscopy and challenges in adequate infrastructure and trained medical personnel to deliver proper diagnostic services in every part of the world, there is an urgent need for targeted interventions with non-invasive and cost-effective procedures to enhance screening uptake rates to mitigate the rising burden of CRC, especially in younger ages [4,17]. Early detection remains pivotal in combating this disease, as it significantly improves patient prognosis and treatment outcomes. This is feasible via a widespread screening for CRC. In recent years, there has been growing interest in the potential of volatile organic compounds (VOCs) as non-invasive biomarkers for CRC detection and monitoring.

VOCs, a diverse group of small molecules emitted from biological processes within the body, offer a promising avenue for the development of sensitive and specific diagnostic tools. Among the various bodily fluids, exhaled breath represents a particularly attractive matrix for VOC analysis due to its non-invasive collection method and direct exposure to systemic metabolic processes.

In this review, we delve into the emerging field of biosensor technology tailored for the detection of VOCs in breath samples as a means to diagnose and monitor CRC. Biosensors, characterized by their ability to transduce biological or chemical signals into measurable outputs, have garnered attention for their potential to revolutionize medical diagnostics. By coupling the selectivity of biological recognition elements with the sensitivity of transducing platforms, biosensors offer a powerful tool for the rapid and accurate detection of CRC biomarkers [18].

**Figure 1:**
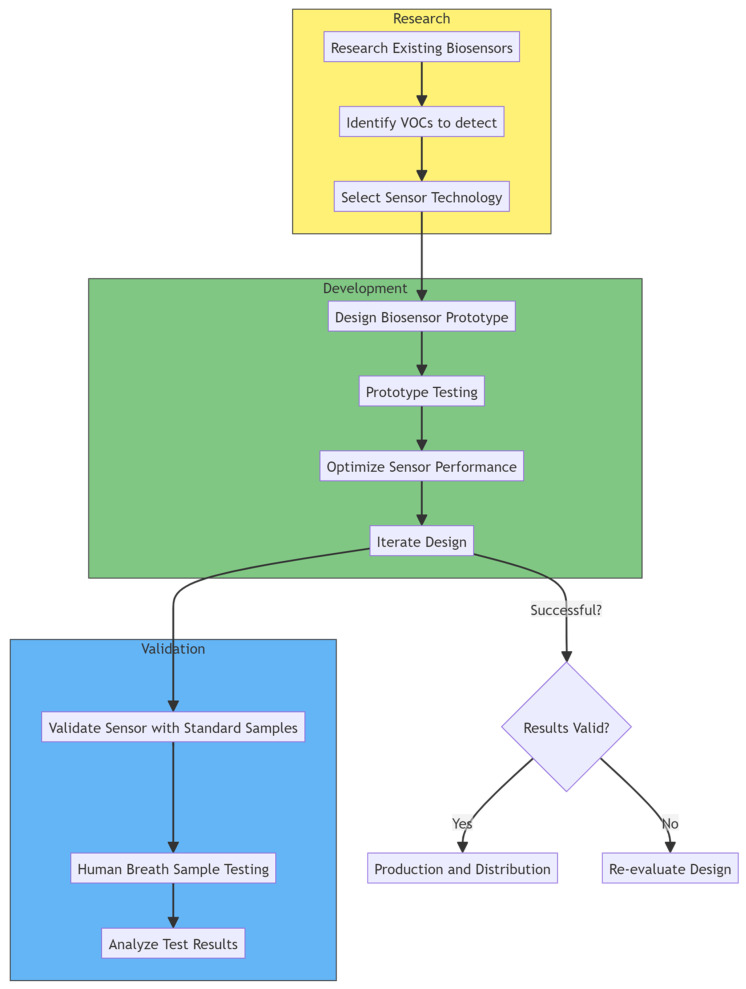
Flowchart of the steps associated with VOC biosensor development.

We aim to provide a comprehensive overview of the current state-of-the-art VOC-based CRC diagnostics, with a specific focus on recent advancements in biosensor design and application. Through a critical analysis of the existing literature and technological innovations, we will explore the challenges and opportunities in harnessing biosensor technology for CRC detection, highlighting key considerations in sensor development, validation, and clinical translation (Figure 1).

By elucidating the intricate interplay between VOCs, CRC pathophysiology, and biosensor technology, this review seeks to contribute to the ongoing efforts toward realizing non-invasive, point-of-care diagnostic solutions for CRC. Ultimately, the integration of biosensor-based VOC detection into clinical practice holds the promise of improving early detection rates, enhancing patient outcomes, and alleviating the burden of CRC on healthcare systems worldwide.

## 2. Overview of Colorectal Cancer and Current Diagnostic Challenges

**Figure 2:**
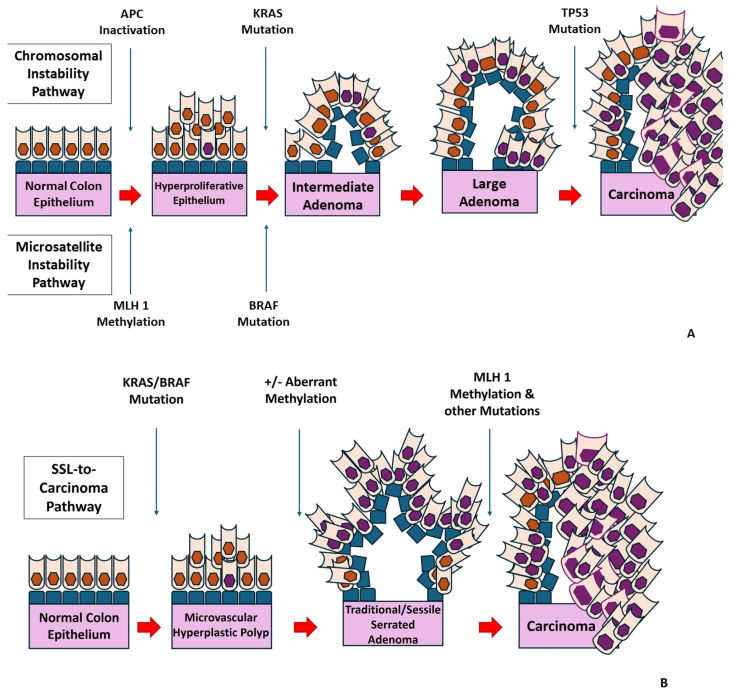
(**A**) CRC is initiated via the adenoma-carcinoma sequence in 70% of cases. (**B**) 25–30% of CRC cases arise through the SSL-to-carcinoma pathways.

Sporadic CRC originates from the epithelial cells lining the colon or rectum and typically develops from non-cancerous precursor lesions, which grow gradually for 10–20 years before sustaining malignant transformation and progressing to invasive carcinomas [19,20,21,22,23]. CRC evolves from cancer stem cells (CSCs), residing in the base of colonic crypts. CSCs following a Darwinian pattern accumulate successive genetic and epigenetic aberrations that inactivate tumor-suppressor genes and activate proto-oncogenes, subsequently conferring selective advantages in terms of cellular survival, proliferation, progression, metastasis, and therapy resistance [24,25,26,27,28]. CRC is a stepwise progression of premalignant lesions; approximately 70% of sporadic CRCs are initiated from adenomatous polyps via the adenoma–carcinoma sequence (Figure 2A), governed by mutation of the tumor-suppressor APC (adenomatous polyposis coli), followed by the acquisition of mutations in proto-oncogene KRAS (Kirsten rat sarcoma virus) and the tumor-suppressor P53 (tumor protein P53; transformation-related protein 53; TRP53), chromosomal, and microsatellite instability (MSI), in increasing adenomas and invasive adenocarcinomas. The remaining 25–30% of CRC arise from sessile serrated lesions (SSLs) through the SSL-to-carcinoma pathways (Figure 2B), associated with mutations in the proto-oncogene BRAF (v-raf murine sarcoma viral oncogene homolog B), MSI, and gene promoter hypermethylation (CpG island methylator phenotype) [7,19,28,29]. Thus, most CRCs are amenable to prevention strategies, offering a window of opportunity to be identified as a precursor, nonmalignant lesions, or in the earliest and highly curable stage.

**Figure 3:**
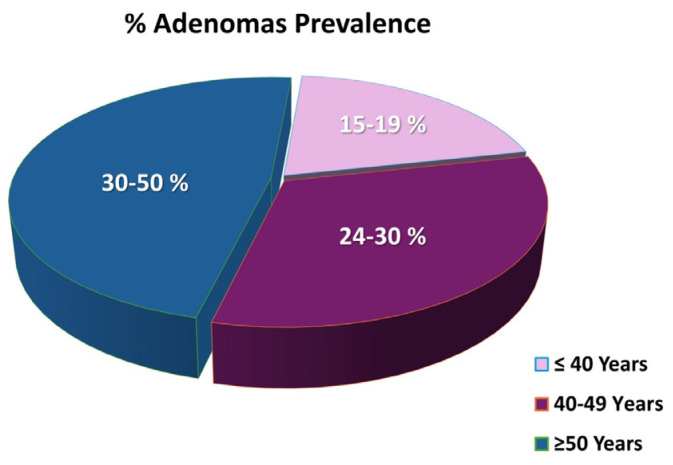
Prevalence of adenomas in the general population.

The advent of high-definition endoscopes, which can detect 1–2 mm lesions, has revealed that adenomas are extremely common in the general population; the prevalence reaches 15–19%, 24–30%, and 30–50% in individuals ≤40 years, 40–49 years, and ≥50 years, respectively [30] (Figure 3). Less than 5–10% of all adenomas harbor malignant potential, and their size, histological type, architectural growth, and dysplastic grade are predictive of their evolution to CRC. The risk of cancerous transformation is 1–3%, 10%, and >40% for adenomatous polyps <1 cm, 1–2 cm, and >2 cm, respectively [19]. In high-grade dysplasia, the risk of invasive carcinoma is 27% [19,31,32].

Adenomatous polyps are histologically classified into tubular, villous, and tubulovillous adenomas. Tubular adenomas account for 70–85% of all adenomas and consist of dysplastic tubular glands, which rarely contain concomitant high-grade dysplasia (HGD). Villous adenomas account for <5% of all adenomas, comprising strands of dysplastic epithelium. They are generally large and sessile, and harbor a high prevalence of HGD or adenocarcinoma progression. Tubulovillous adenomas account for 10–25% of all adenomas and are characterized by a mixture of tubular and villous architecture [19,31,32]. Serrated polyps are a heterogeneous group of colorectal lesions, which include the following: (a) hyperplastic polyps (HPs), which are innocuous lesions and constitute 80–90% of all serrated polyps and 10–15% of all colorectal polyps; (b) sessile serrated adenomas (SSA), which represent 10–25% of all serrated polyps and 3–9% of all colon polyps; (c) traditional serrated adenomas (TSA), which account for 1–2% of serrated lesions and 7–15.9% of all colorectal polyps, and harbor serrated hyperplastic-like architecture in parallel with dysplasia adenomatous or alterations; (d) mixed serrated polyps, with overlapping histologic features of nondysplastic polyps (HPs or SSA) with a dysplastic (TSA or conventional adenomas) [29,33,34]. They may be subtle endoscopically due to their similar color with the surrounding mucosa, flat morphology, mucin cap, and coverage with adherent fecal material [7,33,35,36,37]. SSAs are the predominant precancerous lesions in the serrated class, although some subtypes of HPs, especially large and/or in the proximal colon, which were previously considered benign, may harbor premalignant potential [34,35,37,38,39].

Advanced adenomas, which are high-risk for malignant transformation, are characterized by certain determinants, as assessed by colonoscopy: (i) size, measuring ≥ 1 cm, conferring a 5.2-fold higher risk; (ii) multiplicity ≥ 3 adenomas, and/or SSAs, associated with a 4.3-fold higher risk; (iii) alarming architectural configuration, defined as those with villous morphology or SSAs, especially in the proximal colon, associated with a 7.4-fold higher risk; and (iv) high-risk cytological features, defined as adenomas harboring HGD, conferring a 13.2-fold risk [40]. CRC develops in 1% of adenomas <1 cm, in 10% of adenomas >1 cm and <2 cm, and in 50% of adenomas >2 cm. Villous growth patterns (tubulovillous and villous adenomas) with HGD are significant features of malignant transformation with the likelihood rising to 50% [19,41]. Collectively, the transformation rate of adenomas into CRC is 0.25% per year. Also, even after the removal of the aforementioned adenomas, there is a need for ongoing surveillance, as the risk of future CRC is sustained [19,31,32,42,43,44].

Patients with CRC present with a wide range of signs and symptoms, but the most common are hematochezia (pooled prevalence, 45% [95% CI, 40–50%]), abdominal pain (pooled prevalence, 40% [95% CI, 35–45%]), and altered bowel habits (diarrhea, constipation, tenesmus, abdominal distension, alternating diarrhea and constipation) (pooled prevalence, 27% [95% CI, 22–33%]) with an interval of 4–6 months from symptom onset to CRC diagnosis [14]. Other symptoms include loss of appetite and weight loss, fatigue, anemia, nausea or vomiting, bowel obstruction or perforation, rectal pain, and an abdominal mass [14]. However, colorectal cancer remains largely an asymptomatic disease, until it reaches an advanced stage. Rectal bleeding is a common symptom of both benign and malignant causes, and therefore additional risk factors might be needed to help identify those people who should undergo further investigation by colonoscopy [28].

### 2.1. Current Diagnostic Challenges

Screening aims at reducing CRC incidence and/or mortality by early detection or CRC prevention by detecting and removing precancerous lesions. Despite the availability of various screening methods, the effectiveness of existing diagnostic approaches for CRC is hindered by several limitations. Conventional techniques such as colonoscopy, fecal blood testing, and imaging modalities have notable drawbacks that compromise their widespread adoption and efficacy.

Direct visualization screening tests for CRC include colonoscopy and flexible sigmoidoscopy (FS), in which a camera visualizes the colon, and CT (computed tomography) colonography, which uses X-ray images, and may reveal locoregional lesions and remote metastases. However, suspicious results in FS or CT colonography (CTC) will need further follow-up with colonoscopy to evaluate the more proximal portion of the colon and differentiate between non-cancerous lesions and CRC [13]. Among the direct visualization tests, a colonoscopy every 10 years or CT colonography every 5 years have greater estimated life-years gained than a flexible sigmoidoscopy every 5 years [13,28]. Colonoscopy screening is associated with a 30% (rate ratio, 0.70 [95% CI, 0.66–0.75]) and 32% (rate ratio, 0.68 [95% CI, 0.61–0.76]) CRC incidence and mortality reduction, respectively, preventing an estimated 50 CRC cases (95% CI, 42–58) and 15 (95% CI, 11–19) CRC deaths per 100,000 person years. Colonoscopy has been associated with a long-term reduction in CRC mortality over 20–30 years [5]. FS screening reduces CRC incidence and mortality by 23% (rate ratio, 0.77 [95% CI, 0.74–0.81]) and 24% (rate ratio, 0.76 [95% CI, 0.70–0.83]), respectively, preventing an estimated 37 (95% CI, 30–44) CRC cases and 11 (95% CI, 7–14) CRC deaths per 100,000 person years [45]. Four randomized major clinical trials have demonstrated that a single round of FS is associated with a relative reduction in CRC incidence of 18% (RR, 0.82; 95% CI, 0.75–0.89), and an overall relative reduction in CRC mortality of 28% (RR, 0.72; 95% CI, 0.65–0.80). When considering distal CRC, a relative reduction of 31% (RR, 0.69; 95% CI, 0.63–0.74) in the incidence and 46% (RR, 0.54; 95% CI, 0.43–0.67) in mortality was demonstrated [5]. In 15 years of follow-up, a pooled analysis of the aforementioned trials showed a rate ratio of 0.79 (95% CI, 0.75–0.83) and 0.80 (95% CI, 0.72–0.88) for CRC incidence and mortality, respectively [46].

Direct visualization tests are performed in a clinical setting and necessitate prior bowel preparation with oral laxatives starting the day before the procedure when considering colonoscopy, and a bowel enema on the day of the procedure, sometimes combined with oral laxatives, when considering sigmoidoscopy [6]. Also, during colonoscopy and flexible sigmoidoscopy, sedation or anesthesia is usually required; thus, recovery time is essential [13,28]. Colonoscopy, although invasive and uncomfortable, is considered the gold standard for CRC screening, with a sensitivity for the detection of adenomas larger than 10 mm ranging from 0.89 (95% CI, 0.78–0.96) to 0.95 (95% CI, 0.74–0.99), and a specificity of 0.89 (95% CI, 0.86–0.91) [13]. When considering CTC, although a non-invasive alternative, its results may vary depending on imaging technique and reader experience. Sensitivity for CRC ranges from 0.86 to 1.0 (95% CI range, 0.21–1.0), and for adenomas larger than 10 mm, it is 0.89 (95% CI, 0.83–0.96). Specificity is not determined for CRC, whereas for adenomas it is 0.94 (95% CI, 0.89–1.0) [5,13,47].

Colonoscopy may be complicated with serious sequelae, such as serious bleedings (17.5 events per 10,000 colonoscopies; 95% CI, 7.6–27.5) and perforations (5.4 events per 10,000 colonoscopies; 95% CI, 3.4–7.4) with increasing risk with advancing age. Colonoscopy should generally be avoided if there is a concern for bowel perforation, e.g., active colorectal inflammation (acute diarrhea, toxic megacolon, ischemic colitis, active inflammatory bowel disease, acute diverticulitis, peritonitis), recent colorectal surgery with colonic anastomosis, or bowel injury and repair, symptomatic colon-containing abdominal wall hernia, and symptomatic or high-grade bowel obstruction, or in patients with arrhythmias, recent myocardial infarction, and hemodynamic instability [48]. Other serious complications include cardiopulmonary events due to sedation and infections [13,28]. Bowel preparation may be complicated by dehydration or electrolyte imbalances, particularly in older adults or patients with comorbidities [13,28,49]. Flexible sigmoidoscopy may be complicated with bleedings (0.5 events per 10,000 sigmoidoscopies; 95% CI, 0–1.3) and perforations (0.2 events per 10,000 sigmoidoscopies; 95% CI, 0.1–0.4). CT colonography is not followed by serious complications, except for the exposure to radiation with the radiation dose, ranging from 0.8 to 5.3 mSv, compared with an average annual background radiation exposure of 3.0 mSv per year in the US, 4 mSv in Europe, and 2.4 mSv worldwide [13,49].

An emerging screening modality is colon capsule endoscopy (CCE), which uses an ingestible capsule with a wireless camera to produce images of the mucosa while transitioning along the gastrointestinal tract. The second-generation CCE (CCE-2), a relatively newer technique, has 76.7–86% sensitivity for significant findings (≥6 mm size or ≥3 polyps irrespective of size), dependent on the percentage of colon surface area imaged in regard to the time the capsule was excreted and about 91% specificity [50,51]. However, about one-third of CCEs will eventually lead to a colonoscopy referral, whereas the interpretation of its results requires a trained clinician and often takes more time than traditional colonoscopy [52].

Stool-based tests include the high-sensitivity guaiac fecal occult blood test (hs-gFOBT) and the fecal immunochemical test (FIT), and have decreased CRC mortality by approximately 10–33% in the last decade, when performed every 1–2 years in individuals aged 50–80 years [53,54]. In hs-gFOBT guaiac, the main reagent detects organic heme by oxidation; thus, false-positive results could be yielded by dietary heme in red meat, peroxidase in uncooked fruit and vegetables (e.g., 5–10 g of radish, horseradish, cantaloupe, and cauliflower have a peroxidase activity equivalent to 1 mL of blood) and antioxidants, e.g., vitamins C and E. Also, prior to hs-gFOBT, it is recommended to avoid anticoagulants, non-steroidal anti-inflammatory drugs, and iron [8]. FIT employs antibodies specifically designed to quantify >10–20 μg of human hemoglobin (Hb)/gr in feces (100 ng/mL); dietary and medication restrictions prior to the test are not necessary [5,8]. However, the blood detected in both tests is indicative of CRC or precancerous lesions, and thus, further evaluation is warranted with colonoscopy [13,28,47]. Although stool-based screening is characterized by high compliance, as it is quick and non-invasive without the need for bowel preparation, anesthesia, or sedation, it requires the person to collect samples directly from their feces, which may be unpleasant. Also, dietary and medication restrictions are a prerequisite for the high-sensitivity hs-gFOBT. Although a single stool sample is enough for FIT, for hs-gFOBT, three consecutive samples from separate bowel movements are required [13].

The sensitivity and specificity of hs-gFOBT for the CRC detection range are 0.5–0.75 (95% CI, 0.09–1) and 0.96–0.98 (95% CI, 0.95–0.99), respectively, whereas the advanced adenomas ranges are 0.06–0.17 (95% CI, 0.02–0.23) and 0.96–0.99 [95% CI, 0.96–0.99]), respectively, as demonstrated in randomized trials [5,13]. When considering FIT, performance is dependent on the threshold for a positive result; a threshold of 10 µg/g is associated with a sensitivity of 0.91 (95% CI, 0.84–0.95) for CRC, 0.40 (95% CI, 0.33–0.47) for advanced adenomas, and a specificity of 0.9 (95% CI, 0.86–0.93 for CRC, and 95% CI, 0.87–0.93 for advanced adenomas). A threshold ≥20 µg/g resulted in a sensitivity of 0.71 (95% CI, 0.56–0.83) with a specificity of 0.95 (95% CI, 0.94–0.96) for CRC detection. OC Sensor, which is a quantitative FIT has a sensitivity for CRC detection of 0.74 (95% CI, 0.64–0.83) and a pooled specificity of 0.94 (95% CI, 0.93–0.96), whereas for advanced adenomas its pooled sensitivity is 0.23 (95% CI, 0.20–0.25) and pooled specificity is 0.96 (95% CI, 0.95–0.97). The diagnostic sensitivity of FIT varies, depending on lesion type and location; proximal lesions are not easily detected, as they may arise from serrated polyps, which are flat and less vascular than traditional adenomas, and thus might not bleed [5,13,55].

Stool DNA-FIT testing (mt-sDNA test) incorporates in FIT the detection of multiple cancer DNA biomarkers from cells shed into stools from the lining of the colon and rectum, such as mutant KRAS, APC, and aberrant methylation of BMP3 (bone morphogenetic protein 3), and NDRG4 (N-myc Downstream-regulated Gene 4). It is considered positive if either the FIT or DNA biomarker component is abnormal [8,56]. Its sensitivity and specificity for CRC detection are 0.93 (95% CI, 0.87–1) and 0.84 (95% CI, 0.84–0.86), respectively, whereas advanced adenomas are 0.43 [95% CI, 0.40–0.46] and 0.89 [95% CI, 0.86–0.92] [8,57,58,59,60]. However, its cost is prohibitive for a wide range of screening programs, as according to the 2022 Medicare fees reimbursement for s-DNA-FIT was USD 509, whereas hs-gFOBT was USD 4.38 and USD 18.05 for FIT [8]. Also, evidence concerning longitudinal follow-up of abnormal findings after a negative colonoscopy is insufficient [59].

The U.S. Preventive Services Task Force (USPSTF) recommends screening initiation: (a) at 45 years of age for average-risk patients, due to recent high early-onset incidence; (b) at 40 years of age or 10 years earlier than the age at diagnosis of the youngest affected with CRC or advanced adenoma first-degree relative, whichever comes first; and (c) as early as adolescence for individuals with underlying risk factors, e.g., cancer syndromes (Lynch syndrome, Peutz–Jeghers syndrome, familial adenomatous polyposis, Gardner syndrome, Turcot syndrome, hereditary non-polyposis colorectal cancer, juvenile polyposis), inflammatory bowel diseases (Crohn’s disease, ulcerative colitis), and radiation therapy to the abdomen, pelvis, or spine at doses of ≥ 30 Gy for a previous childhood cancer [8,48,61]. Furthermore, in the average-risk individuals, it is suggested to repeat colonoscopy every 10 years, which is expected to reduce CRC incidence, and mortality by 58% and 64%, respectively [48]. Subjects with a positive family history should receive a colonoscopy every 5 years. In average-risk individuals, yearly fecal occult blood testing (FOBT) and flexible sigmoidoscopy (FSIG) every 3 years are also accepted methods of screening for CRC [48].

A population-based cohort study of 186,046 patients from 21 medical centers demonstrated that the cumulative incidence of CRC, at 10-year follow-up after colonoscopy were 0.39%, 0.44%, and 1.12% in the null, low-risk, and high-risk adenoma groups, respectively, and at the end of a 14-year follow-up were 0.51%, 0.57%, and 2.03% in the aforementioned groups, respectively. The high-risk adenoma group exhibited a higher risk of CRC (hazard ratio [HR], 2.61; 95% CI, 1.87–3.63) and related death (HR, 3.94; 95% CI, 1.90–6.56), whereas the low-risk adenoma group did not yield a significant increase in risk of CRC (HR, 1.29; 95% confidence interval, 0.89–1.88) or related death (HR, 0.65; 95% CI, 0.19–2.18), during a median follow-up time of 8.1 years [62,63]. Thus, there is a need for post-polypectomy colonoscopy surveillance to achieve better survival benefits for early detection of interval cancers, which usually occur in the first 3–5 years after the index colonoscopy with polypectomy, depending on the number and the size of adenomatous polyp(s), villous component and high-grade dysplasia in the polyp, and the presence of serrated lesions or serrated polyposis syndrome [48]. USMSTF guidelines recommend that patients with 1–2 tubular adenomas < 1 cm and sessile serrated polyp(s) < 1 cm with no dysplasia should be followed at a 10- and 5-year interval, respectively, whereas patients with a high-risk adenoma, sessile serrated polyp(s) ≥ 1 cm, or with dysplasia or serrated adenoma should repeat colonoscopy in 3 years [48,63]. Similarly, the European Society of Gastrointestinal Endoscopy (ESGE) and consensus guidelines, jointly commissioned by the British Society of Gastroenterology (BSG), the Association of Coloproctology of Great Britain and Ireland (ACPGBI), and Public Health England (PHE) suggest for the low-risk group (complete removal of 1–4 adenomas < 10 mm with low-grade dysplasia, irrespective of villous components or serrated polyps < 10 mm in size without dysplasia) follow-up colonoscopy at 10 years and a 3-year repetition of surveillance colonoscopy in high-risk individuals (complete removal of at least one adenoma ≥ 10 mm or with high-grade dysplasia, or ≥ 5 adenomas, or serrated polyps ≥ 10 mm in size or containing any grade of dysplasia) [30,63]. ESGE also recommends a repeat colonoscopy in a 3–6-month interval, following endoscopic piecemeal resection of polyps ≥ 20 mm, followed by a surveillance colonoscopy at 12–18 months to detect late recurrence [30,48,63]. Given the lower incidence of CRC among patients ≥75 years [HR for CRC 0.06, 95% CI 0.02–0.13; *p* < 0.001] and the high risk of post-procedure hospitalization [adjusted OR 1.28, 95% CI 1.07–1.53; *p* = 0.006], and that the severe adverse effects of colonoscopy are associated with age and comorbidities, it is recommended to avoid post-polypectomy surveillance routinely on patients over 75 years, or when underlying diseases indicate that life expectancy is likely to be less than 10 years [30,64]. CTC, although attractive in colonic surveillance, is not recommended for surveillance after resection of premalignant colorectal polyps, as the radiation hazard is outweighed by its potential benefits [30]. Similarly, FIT is also not recommended for post-polypectomy surveillance, due to insufficient evidence; annual FIT could miss 40–70% of advanced adenomas and 30–40% of CRCs [63,65]. However, in “intermediate-risk” patients (3–4 adenomas < 1 cm, or 1–2 adenomas with one ≥ 1 cm) annual FIT with low threshold levels for fecal Hb (10 μg/g) could reduce colonoscopies by 71% and yields high sensitivity for CRC detection (three cumulative tests: sensitivity of 91.7% [95% CI 73.0–99.0] and specificity of 69.8% [95% CI 68.5–71.1]) [63].

When considering CRC post-resection colonoscopy surveillance for an early diagnosis of CRC recurrence at an anastomotic location or missed, new-onset, or incompletely resected lesions at non-anastomotic locations, the recommendations are conflicting [30,63,64,66,67]. However, the American Cancer Society (ACS) recommends a follow-up colonoscopy in the first year, after curative resection, for early detection of metachronous CRCs and advanced adenomas, or anastomotic recurrence of the initial primary cancer. In these patients, there is a 1.5–2-fold increase in metachronous CRC compared with the general population, corresponding to a 1–2% long-term risk [68]. Follow-up colonoscopies are repeated at 3- and 5-year time intervals if the findings are negative for CRC [30,63]. A large cancer registry cohort study from the Netherlands of sporadic CRC patients demonstrated a mean annual incidence of metachronous CRC at 0.3% and a cumulative incidence of 1.1% at 3 years, 2.0% at 6 years, and 3.1% at 10 years. The presence of synchronous CRC missed at index colonoscopy was verified as a significant risk factor for metachronous CRC [relative risk (RR) 13.9, 95% CI 4.7–41.0], especially in the first 3 years of follow-up [64,68]. A recent systematic review and meta-analysis demonstrated a low absolute risk for metachronous CRC ranging from 0.63% to 0.74% (95% CI, 0.47–1.09%) at 6–36 months, which significantly decreased to 0.28–0.45% (95% CI, 0.14–0.70%) during 37–120 months of follow-up [67]. When considering anastomotic lesions, the risk reached 1.7–1.23% at 6–24 months post-operatively (95% CI, 0.74–2.8%), whereas it further decreased after 24 months to 0.3–0.93% (95% CI, 0.14–1.6%) during 24–48 months of follow-up [67]. 

In terms of the most cost-effective screening modality, as assessed by direct healthcare costs and indirect costs in the USA (e.g., time lost from work), and life-years gained (LYG) after age 45 years, colonoscopy with a mean cost of USD 9037 per person was the ideal choice, yielding an ICER (Incremental Cost-effectiveness Ratio)/LYG of USD 28,071, and 35.67 LYG. FIT was associated with 35.62 LYG, and sDNA-FIT with 35.64 LYG [69]. However, there is a steep increase in colonoscopies, owing to the expansion of population screening programs, and the surveillance burden of at-risk patients (syndromes associated with CRC, previously identified CRC, or advanced adenoma). In a UK colonoscopy audit, 65.4% of colonoscopies were undertaken for diagnostic purposes, 9.7% of procedures during the implementation of the National Bowel Cancer Screening Program of average-risk individuals of the general population, and 17.7% of colonoscopies were performed for surveillance of a known high-risk group. Interestingly, CRC and colorectal polyps were diagnosed in 4.1% and 27.5% of colonoscopies, whereas 41.8% of examinations were normal [70].

Collectively, we should consider the cost-effectiveness of wide screening programs of average-risk individuals in the general population or the early and frequent colonoscopic surveillance after CRC, and/or polyp(s) resection, the procedural distress, especially in younger individuals, and the adverse effects, which augment with age and comorbidities, with additional concerns regarding radiation exposure, cost, and availability of the other direct visualization tests. Also, the stool-based tests yield varying sensitivity, and specificity, and need a follow-up endoscopy. Thus, there is a growing need for the provision of an alternative appropriate screening service as part of a risk-stratified approach, characterized by high-quality, high sensitivity and specificity for CRC, and its precursor lesions, safety, low cost, and minimal invasiveness (low burden and risk of complications), ensuring compliance, even in younger ages, sustainability over time, and equity of access worldwide. Such an approach would encourage broad population screening, prioritize patients in which colonoscopy is warranted, reduce ‘wasted’ resources, and decrease waiting times and healthcare expenses [28,71,72].

### 2.2. The Imperative for Non-Invasive and Accessible Diagnostics

**Figure 4:**
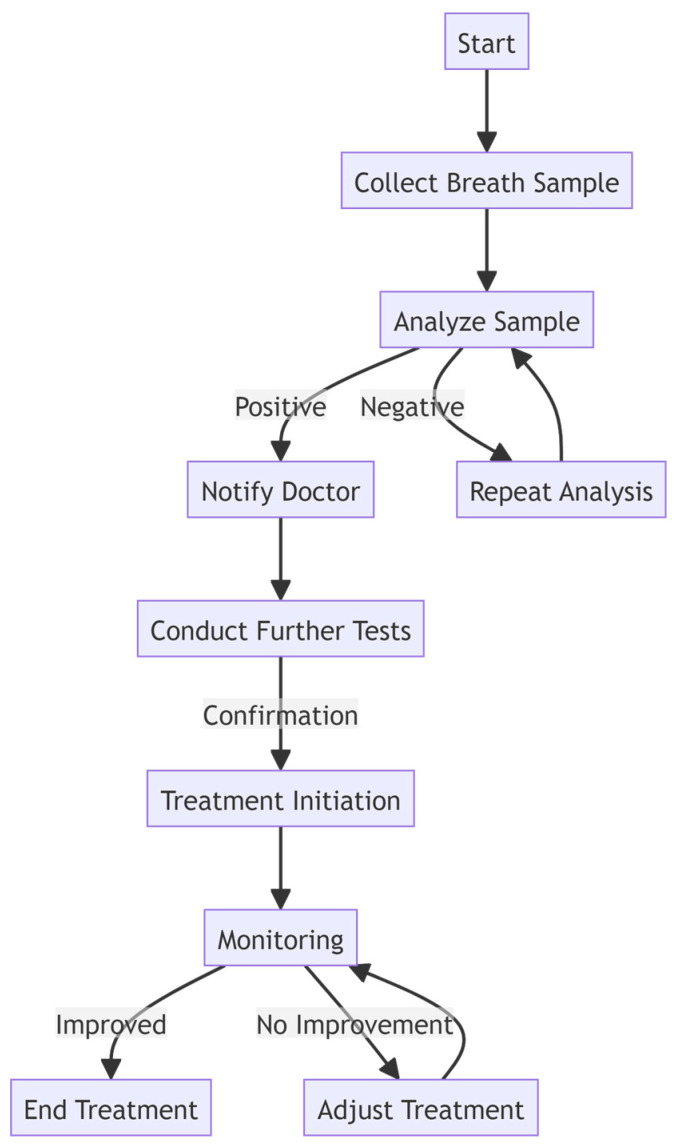
Flowchart of the process from collecting the sample to treatment. By using a POC biosensor to analyze samples and detect VOCs in patients’ breath samples, the process becomes quicker, resulting in faster treatment for the patients.

Although conventional screening modalities have played a crucial role in diagnosing CRC, given their limitations, there is an urgent need for non-invasive and more accessible diagnostic approaches for CRC (Figure 4). Such methods would facilitate early detection in primary and latent stages, improve patient compliance, and ultimately reduce CRC-related morbidity and mortality. Emerging technologies, including biosensors capable of detecting volatile organic compounds (VOCs) associated with CRC, hold promise as adjuncts to existing screening strategies. By leveraging advancements in molecular biology and sensor technology, these innovative approaches offer the potential for earlier detection, risk stratification, and personalized management of CRC patients. VOC detection could serve as a non-invasive test, selecting individuals at a higher risk of advanced adenomas or CRC for colonoscopy and relieving low-risk patients of the burden and risk of colonoscopy [5]. Biomarker-based approaches for CRC detection hold significant promise, yet face formidable challenges. These obstacles include the need for rigorous validation, navigating CRC’s complex heterogeneity, ensuring affordability and accessibility, minimizing both false positives and negatives, standardizing biomarker assays, and ensuring effective follow-up and treatment options. Despite these challenges, ongoing scientific research, technological innovations, and collaborative efforts suggest the potential for a transformative shift in CRC screening, diagnosis, and treatment strategies. This evolution hinges on the integration of biomarkers as a crucial element of routine clinical practice and volatile organic compounds (VOCs) as non-invasive biomarkers combined with biosensors, and nanotechnology-driven innovations appear to have great potential in CRC diagnostics.

## 3. Volatile Organic Compounds as Biomarkers in Colorectal Cancer

VOCs are biomarkers, along with proteins, DNA, and RNA, which can be used as indicators to detect alterations in the human body. These biomarkers can be used to differentiate between pathological and normal conditions, to enable objective measurement in the case of cancer or other specific diseases, and to predict the possible treatment outcome [73,74]. VOCs are small, carbon-based molecules that are emitted as gasses from biological processes within the human body.

Biomarkers can generally be classified into two main groups: invasive and non-invasive. In the case of invasive biomarkers in CRC, there is blood and endoscopy. In the first case, proteins originating from cancer cells can be detected [75], while endoscopy is used to identify idiopathic symptoms [76]. Non-invasive biomarkers include urine, feces, sweat, breath, and nipple aspirate fluid, depending on the type of cancer [77,78,79,80,81].

Ever since Linus Pauling revealed in 1971 an estimated number of 250 VOCs in human breath and urine [82], the science of detecting human diseases through exhaled breath has grown exponentially, with new sensors being developed. Chromatography–mass spectrometry (GC-MS) has been used to identify most of the individual VOCs mentioned in relevant studies. This technique is considered to be the gold standard, as it is easily reproducible and has high sensitivity as a technique and robustness for individual VOCs [83].

Volatile organic compounds (VOCs) are emerging as promising biomarkers for CRC due to their unique signatures associated with the disease. They are produced through various metabolic processes occurring within the human body. These compounds can originate from endogenous sources, such as cellular metabolism, gut microbiota activity, and oxidative stress reactions. Additionally, exogenous factors, including diet, environmental exposures, and medications, can influence VOC production [84]. Moreover, VOCs can also cause oxidative stress and inflammation, which contribute to different types of cancer [84]. They have also been reported to cause some biological reactions, such as apoptosis or cell growth, leading to the development of tumors and metastasis. VOCs are transported via blood circulation and are expelled through breath, urine, feces, and skin. They can be analyzed and sampled for various diagnostic purposes. In this review, we are going to mainly focus on VOCs expelled through breath and their potential to serve as CRC biomarkers offering non-invasive, sensitive, and cost-effective diagnostic tools.

The alteration of VOC profiles in CRC is multifactorial and reflects the underlying pathophysiological changes associated with tumorigenesis and tumor progression. Metabolic dysregulation, inflammation, oxidative stress, and alterations in gut microbiota composition contribute to the production and release of specific VOCs associated with CRC. These VOCs may arise from aberrant cellular metabolism, tumor–host interactions, and the tumor microenvironment. Furthermore, CRC-specific genetic and epigenetic alterations may influence VOC production and metabolism.

### 3.1. Specific VOC Signatures Associated with CRC

Numerous studies have identified distinct VOC signatures associated with CRC, characterized by changes in the abundance and composition of specific compounds (Table 1). Elevated levels of aldehydes and ketones have been reported to be primarily expressed in all cancers and in CRC patients compared to healthy controls. Moreover, unique VOC profiles have been observed at different stages of CRC development, suggesting the potential for disease staging and prognostication.

Aldehydes are associated with CRC, among other types of cancer [85,86]. Compared to normal cells, the metabolic processes of cancer cells produce abnormal organic compounds allowing for changes in concentration in exhaled breath to be associated with cancer biomarkers in gastrointestinal cancer patients [30]. Janfaza and his team developed an analysis of the Cancer Odor Database (COD) [87] that indicates the contribution of specific VOCs to particular types of cancer. These VOCs have the potential to act as biomarkers according to their research [87,88]. Among the aldehydes that are associated with CRC, as well as with other types, hexanal, heptanal, and nonanal aldehydes are the most common to be detected in exhaled breath of cancer patients [88]. Compared to the profiles of healthy subjects, some lipids, both saturated and unsaturated, are observed at altered levels due to the composition of membrane lipids in cancer cells [89]. The production of some aldehydes may be promoted by the increased concentrations of unsaturated fatty acids through lipid peroxidation [85,90], and this is the reason for the differentiation of the metabolism of aldehydes in cancer cells compared to normal cells [91].

Ketones are derived from external factors, such as diet, and can be affected by these factors. However, in different types of cancer, the production of ketones is initiated by a typical mechanism of increasing long-chain fatty acid (LCFA) oxidation, which increases the ketone body synthesis in the liver’s mitochondria [92,93]. The initial stage of the fatty acids’ catabolism is β-oxidation, which breaks down fatty acids with the use of ΝADH and FADH2, and other electron transport chain factors, followed by the production of acetoacetyl-CoA [94]. Ketone bodies are regulated differently in normal tissue than in tumor tissues. They can be degraded into acetyl-CoA to enhance cell viability by producing energy as they enter the tricarboxylic acid (TCA) cycle [95,96].

Due to the different mitochondrial structures of cancer cells, the oxidative stress of ketone bodies may be increased through the TCA cycle and, moreover, inhibition of the antioxidant system pathway occurs due to the increased ROS. The formation of ketone bodies is a direct result of acetoacetyl and they are released into the plasma and transported through the blood vessels to the lungs and exhaled afterward [97,98]. Four ketones are considered to be cancer biomarkers 2-nonanone, 3-heptanone, cyclohexanone, and 4-heptanone, but there are certain limitations to their use in detecting cancer [99,100].

Besides aldehydes and ketones that increase in CRC patients, other VOCs that have been mentioned to alter include alcohols and indole, which also appear to increase [101,102,103,104], and benzene ethyl, which is decreased [102].

According to Wang and their team [105], higher levels of multiple VOCs were detected in the breath of CRC patients other than the abovementioned cyclohexanone, including dimethyldecane, dodecane, 4-ethyl-1-octyn-3-ol, ethylaniline, cyclooctylmethanol, trans-2-dodecen-1-ol, and 3-hydroxy-2,4,4-trimethylpentyl 2-methylpropanoate, and significantly lower levels of 6-t-butyl-2,2,9,9-tetramethyl-3,5-decadien-7-yne. The team of Altomare more recently [106] studied the breath print of CRC patients and healthy individuals and found fourteen VOCs to have a significant ability to detect patients with CRC (tetradecane, ethyl-benzene, methylbenzene, acetic acid, 5,9-undecadien-2-one, 6,10-dimethyl (E), decane, benzaldehyde, benzoic acid, 1,3 bis(1-metiletenil) benzene, decanal, unidentified compound T22_75, dodecane, 2-ethyl-1-hexanol, and ethanone, 1[4-(1-methylethenyl)phenyl]) with their model resulting in a 93% true predictive value for this type of cancer and with the reliability of the breath analysis maintained with 9% specificity and 86% sensitivity, regardless of the cancer stage.

**Table 1:** Specific VOC signatures associated with CRC through exhaled breath samples.

Volatile Organic Compound	References
cyclohexanone dimethyldecane dodecane 4-ethyl-1-octyn-3-ol Ethylaniline cyclooctylmethanol trans-2-dodecen-1-ol 3-hydroxy-2,4,4-trimethylpentyl 2-methylpropanoate	Wang et al. [105]
tetradecane, ethyl-benzene, methylbenzene, acetic acid, 5,9-undecadien-2-one, 6,10-dimethyl(E), decane, benzaldehyde, benzoic acid, 1,3 bis(1-metiletenil) benzene, decanal, unidentified compound T22_75, dodecane, 2-ethyl-1-hexanol and ethanone, 1[4-(1-methylethenyl)phenyl]	Altomare et al. [106]
acetone ethyl acetate 4-methyl-octane ethanol	Amal et al. [107]
1,1′-(1-butenylidene)bis benzene 1,3-dimethyl benzene 1-iodo nonane [(1,1-dimethylethyl)thio] acetic acid 4-(4-propylcyclohexyl)-40-cyano[1,10-biphenyl]-4-yl ester benzoic acid 2-amino-5-isopropyl-8-methyl-1-azulenecarbonitrile	Peng et al. [108]

### 3.2. Techniques Used for Breath VOCs

Solid-phase microextraction methods including gas chromatography/mass spectrometry (SPME-GC/MS) were used by Wang et al. [105], along with the statistical methods of principal component analysis (PCA) and partial least squares discriminant analysis (PLS-DA) for the final data processing. Gas chromatography–mass spectrometry was also used by Altomare et al. [106] and the discrimination of each VOCs’ ability to detect CRC was carried out by the use of ROC curve analysis and the cross-validation of the results of their research was made by applying stepwise logistic regression analysis (Table 2).

These pattern-based techniques were also used in the study of Van Keulen [109], who used an electronic nose, a portable device with three metal-oxide sensors, each with different material properties. CG-MS has the advantage of high efficiency, but the high cost and low manageability are significant disadvantages. Amal et al. used CG-MS and e-nose made by cross-reactive nano-arrays to analyze their exhaled breath samples from CRC patients and healthy volunteers [107], and their results revealed four compounds that had a significant difference in the CRC group compared to the healthy group, with acetone and ethyl acetate being higher in the CRC patients while ethanol and 4-methyl-octane are lower than the control group. Good discrimination between CRC and control groups, as well as with other cancer-type groups, was achieved with the use of nano-array technology.

A metabolomic breath analysis was conducted by Peng et al. [108] (Table 2) using a 14-nanosensor array that was tailor-made and based on organically functionalized gold nanoparticles (GNPs). SPME-GC/MS analysis was again used in this study for the identification of representative and suitable VOCs to distinguish CRC and the GNP array appeared to have high discriminant ability in identifying cancer patients from healthy controls.

### 3.3. Breath Sampling and Analysis Technologies

A non-invasive technique for collecting exhaled breath samples that may be used to evaluate a variety of disease parameters is called exhaled breath condensation. Airway Lining Fluid (ALF) droplets that have evolved by turbulence from all lung compartments and are held in a matrix of condensed moisture from the breath compose the Exhaled Breath Condensate (EBC). These droplets contain numerous biomarkers including DNA, RNA, mRNA, proteins, metabolites, and volatile organic compounds (VOC).

There are commercial solutions that capture these droplets and present them as a fluid pool [114,115,116]. From there, the samples can be easily extracted and further analyzed. The breath is collected from the subject, who breathes normally into the device and the breath condensate is gathered into a transportable cartridge. This allows for easy integration of the sample tube into existing studies and allows a large amount of data to be collected with ease from various environments. Microfluidic platforms can later be used for sample distribution and analysis.

## 4. CRC Diagnosis and Nanotechnology

Early-stage cancer cases or precancerous lesions might be missed by conventional methods, offering a late diagnosis and treatment to patients. It is significant to acquire the discriminatory power to distinguish CRC patients from healthy individuals and, therefore, imperative to increase the sensitivity and specificity of CRC screening methods [117]. Timely and successful interventions even at a precancerous stage are crucial and high specificity is important in order to minimize false-negative or false-positive results [118].

### 4.1. Nanoparticles

The rapidly evolving field of nanotechnology offers novel diagnostics for CRC. Nanomaterials with their unique properties and capabilities enable early diagnosis and personalized treatment options with the sensitive and specific detection of VOCs and other biomarkers. The unique physicochemical properties of nanomaterials enable different biomarkers to be captured efficiently and, moreover, may provide multiplexed analysis in a single assay. Customization for breath VOCs is possible, along with other diverse sample types [119]. Some of the nanoparticles that can be utilized are quantum dots (QDs), gold nanoparticles (AuNPs), and magnetic nanoparticles.

Quantum dots (QDs) have unique optical characteristics and are known for their photostability, which enables multiplexed imaging capabilities [120]. Their ability to conjugate with ligands and bind with target molecules enables precise imaging of the target, making QDs significant tools for the advancement of sophisticated imaging modalities in the research and diagnosis of CRC. Carbon-based nanoparticles include carbon nanotubes, graphene, and carbon dots and offer multiple advantages for CRC diagnosis. A notable characteristic is their biocompatibility compared to gold nanoparticles and QDs. Also, their high electrical conductivity and large surface area make them ideal to be functionalized with specific biomolecules [121]. Carbon-based nanoparticles can be integrated with impedance and electrochemical biosensors and may be used in point-of-care (POC) platforms. An example of this can be given by Yan and their associates, who used carbon nanoparticles for the detection of lymph node metastasis in the first two stages of CRC [122], with results demonstrating that these nanoparticles effectively traced lymphatic drainage and accurately identified sentinel lymph nodes in stage 1 and stage 2 CRC. Several advantages for the diagnosis of CRC offer lipid-based nanoparticles as well. These nanoparticles comprise phospholipids or cholesterol and can be functionalized with specific ligands or antibodies to selectively recognize CRC biomarkers [123].

Various types of nanomaterials have been utilized to improve chemiresistive sensors and their sensitivity properties, as mentioned. The main problem, though, is their wide response to a variety of gas phase analytes, which therefore requires an array of sensors to be able to distinguish between different VOCs [124]. This low sensitivity issue can be addressed with the use of molecularly imprinted polymers (MIPs), polymeric nanocomposites that have been proposed to be used as sensitive film [110,125]. A chemiresistor sensor for the detection of toluene vapor was developed by Alizadeh and Rezaloo based on MIPs and carbon black powder and it showed a significant response to toluene [110] (Table 2). Similarly, a mixture of MIPs and other nanomaterials has been used to develop a sensor for nitrobenzene vapor detection [125]. A sensor that could lead to detecting colorectal cancer VOCs was synthesized by Janfaza and their team, who developed hexanal-imprinted polymer nanoparticles via precipitation polymerization for recognition of hexanal along with a new sensor based on MIP nanoparticles and multiwalled carbon nanotubes [111] (Table 2). Their research showed that the detection of aldehydes is instrumental in diagnosing CRC and that the selective sensing of particular gas phase analytes can be achieved with the use of MIPs in chemiresistors.

However, there are limitations requiring careful scientific consideration in these nanotechnology advancements. Nanoparticles that are employed in diagnostic applications must be checked for biocompatibility and possible toxicity. Their safety profile is imperative to be ensured and comprehensive assessments should be implemented. Another limitation could be the cost of mass production of such diagnostic modalities, along with technical challenges. Durability and long-term stability should be meticulously scrutinized in order for the diagnostic platforms to be reliable.

### 4.2. Microfluidics

A foundation for lab-on-a-chip devices can be provided by microfluidic technologies and miniaturized systems offering numerous advantages, such as reduced reagent consumption, enhanced sensitivity, portability, reduced sample quantity, and accelerated reaction times [126]. It is a noteworthy initiative to incorporate biosensing for CRC detection into microfluidic devices. In this context, colorimetric and electrochemical biosensing modalities are very relevant. Microfluidics has greatly improved detection and diagnostic methods, triggering a significant transformation in the field of CRC by providing a non-invasive avenue for the detection of biomarkers associated with colorectal cancer.

Furthermore, microfluidic systems, when combined with paper-based systems, gain more powerful and beneficial features. Paper-based microfluidic analytical devices (μPADs) have been developed for POC testing and offer multiple advantages, including low cost, the ability to perform multiple tests, and the ability to work without power thanks to the capillary action liquid transfer [127].

The presence and characterization of malignancy-related biomarkers for patients with CRC is important for the understanding of the biological pathways and activities involved and of the pharmacological response to therapeutic interventions. Biomarkers in general, and VOCs in particular, can offer valuable clinical insights to clinicians regarding the stage of the cancer, allowing for informed decisions regarding the necessary treatment options [128]. μPADs for cancer biomarker detection are very promising for improving cure rates and quality of life, and for minimizing treatment costs as they overcome operational difficulties by working on a simple principle based on a continuous process due to the flow through the microfluidic channels [127].

Ghazi et al. presented a study in which they enhanced the selectivity of microfluidic gas sensors towards different VOCs, including hexanal, which is a biomarker for CRC. Their team used 3D printing and coating with Graphine Oxide (GO) to introduce microfeatures and nanofeatures on the microchannel’s surfaces [112] (Table 2). The study by Paknahad and their team presented two microfluidic-based gas detectors with different hydrophobicity, each fabricated with a different channel coating [113] (Table 2). These detectors were used for ketones, alcohols, and alkanes and the results were qualitatively and quantitatively compared, showing that non-polar analytes exhibit a greater difference in the solid–liquid interface than the polar ones, according to a comparison of the surface tensions of the two channels.

## 5. Discussion and Conclusions

### 5.1. Future Perspectives

Certain volatile organic compounds are related to specific types of cancer and can be utilized for the distinction between healthy samples and cancer patients. Ketones and aldehydes mentioned in this review have been shown to be identifiable in human breath a few minutes after being released from tissues due to their solubility in blood [80,129]. Ten VOCs have been associated with CRC, in addition to hydrocarbons and aromatics that originate from exogenous factors. Among these biomarkers, various studies revealed through different methods that hexanal and 3-heptanone are the most reported to be among cancers of the gastrointestinal system [130,131,132].

The research presented in this study has unveiled significant cancer-related dimensions within volatile organic compound (VOC) profiles. Within the medical realm, biomarkers stand as pivotal elements in the shift toward a personalized healthcare paradigm, emphasizing prevention and treatment tailored to individual experiences and statistical data, transcending conventional collective diagnostic approaches [133,134]. The global biomarker market shows consistent growth, paralleling the expansion of biomarker research across various diseases alongside advancements in medical technology [135]. Ongoing efforts to develop more sophisticated biomarkers hold promise for enhancing their utility in clinical settings [136,137,138]. Despite strides in medical technology, the challenge persists in detecting cancer symptoms before reaching fatal stages, compounded by the financial barriers hindering patient access to medical tests in the absence of insurance coverage.

This article provides a comprehensive review of the metabolization of aldehydes and ketones in colorectal cancer, underscoring the invaluable insights VOCs offer into the biochemical processes of cancer cell metabolism. The thorough analysis of identifiable VOCs in exhaled breath presents an avenue to alleviate the burden of invasive medical tests for patients, potentially enabling early cancer detection and post-surgery prognosis prediction at minimal cost.

Analyzing vast quantities of quantitative data and predicting cancer based on VOC parameters is part of the future with biosensors. Leveraging machine learning and deep learning algorithms, scientific approaches enable precise cancer detection using VOCs from exhaled breath, mitigating the influence of environmental factors and yielding accurate prediction models [139]. The accumulation of extensive data over fifty years on the relationship between VOCs and cancers has laid the groundwork for their systematic analysis, facilitated by advancements in data processing technologies [140]. The integration of bioelectronic and olfactory-receptor-based sensors as primary transducers showcases remarkable sensitivity, simplicity, and affordability, offering promising alternatives to conventional diagnostic instruments [111].

### 5.2. Conclusions

In conclusion, the investigation of VOCs from exhaled breath represents a frontier of innovation with significant potential for identifying biomarkers in gastrointestinal cancers, making further studies to amass adequate data necessary. Notably, exogenous factors, particularly physical activities and smoking, can influence VOC patterns, complicating their interpretation [141]. Numerous studies have identified distinct VOC signatures associated with CRC [142,143,144] and, while certain VOCs, like acetone, may show promise as cancer biomarkers, limitations arise from their susceptibility to fluctuations during various activities, rendering them inadequate for reliable biomarker status. Similarly, uncertainties persist regarding the origins of many VOCs, such as 4-heptanone, precluding their recommendation as biomarkers [145]. Nevertheless, leveraging advanced instrumentation and developing new biosensors accompanied by big data analytics holds promise for unraveling the complexities surrounding VOCs in colorectal cancer, potentially revolutionizing cancer prevention and treatment strategies in the future.

## Figures and Tables

**Table 2:** Techniques used to detect VOCs either from patient samples (1–5) or for the development of different sensors (6–9).

	Technique	Sample Used	CRC-Related VOCs Identified:	Reference
1	Solid-phase microextraction gas chromatography/mass spectrometry (SPME-GC/MS)	Exhaled breath from patients and healthy subjects	3-hydroxy-2,4,4-trimethylpentyl 2-methylpropanoate, cyclohexanone, 2,2-dimethyldecane,cyclooctylmethanol, dodecane, 4-ethyl-1-octyn-3-ol,ethylaniline, trans-2-dodecen-1-ol, and 6-t-butyl-2,2,9,9-tetramethyl-3,5-decadien-7-yne	Wang et al. [105]
2	Gas chromatography–mass spectrometry	Exhaled breath from patients and healthy subjects	tetradecane, ethyl-benzene, methylbenzene, acetic acid, 5,9-undecadien-2-one, 6,10-dimethyl (E), decane, benzaldehyde, benzoicacid, 1,3 bis(1-metiletenil) benzene, decanal, unidenti-fied compound T22_75, dodecane, 2-ethyl-1-hexanoland ethanone, 1[4-(1-methylethenyl)phenyl]	Altomare et al. [106]
3	E-nose technology	Exhaled breath from patients and healthy subjects		Van Keulen et al. [109]
4	GC-MS and cross-reactive nanoarrays in combination with pattern recognition methods	Exhaled breath from patients and healthy subjects	Ethanol, acetone, ethyl acetate, -methyl octane	Amal et al. [107]
5	Functionalized gold nanoparticles (GNPs)	Exhaled breath from patients and healthy subjects	¼1,10-(1-butenylidene)bis benzene; ¼1,3-dimethyl benzene; ¼1-iodo nonane; ¼[(1,1-dimethylethyl)thio] aceticacid; ¼4-(4-propylcyclohexyl)-40-cyano[1,10-biphenyl]-4-yl ester benzoic acid; ¼2-amino-5-isopropyl-8-methyl-1-azulenecarbonitrile	Peng et al. [108]
6	Molecular imprinted polymers (MIPs) chemiresistor sensor	-	toluene	Alizadeh and Rezaloo [110]
7	Molecular imprinted polymer nanoparticles (MIPs) chemiresistive sensor	-	hexanal	Janfaza et al. [111]
8	Microfluidic gas sensor— 3D printing and coating with Graphine Oxide (GO)	-	methanol, ethanol, pentanol, hexanal, toluene	Ghazi et al. [112]
9	Microfluidic-based gas detector—3D-printed with a metal oxide semiconductor (MOS) gas sensor	-	acetone, methanol	Paknahad et al. [113]
